# Genetic and Non-genetic Workup for Pediatric Congenital Hearing Loss

**DOI:** 10.3389/fped.2021.536730

**Published:** 2021-03-22

**Authors:** Ryan Belcher, Frank Virgin, Jessica Duis, Christopher Wootten

**Affiliations:** Division of Pediatric Otolaryngology, Vanderbilt Department of Otolaryngology - Head and Neck Surgery, Monroe Carell Jr. Children's Hospital, Nashville, TN, United States

**Keywords:** genetic, hearing loss, congenital hearing deficit, hearing problems, genetic algorithm

## Abstract

Hearing loss is one of the most common concerns for presentation for a geneticist. Presentation prior to the age of one (congenital hearing loss), profound sensorineural hearing loss (SNHL), and bilateral hearing loss are sensitive and should raise concern for genetic causes of hearing loss and prompt referral for genetic testing. Genetic testing particularly in this instance offers the opportunity for anticipatory guidance including possible course of the hearing loss over time and also connection and evaluation for additional congenital anomalies that may be associated with an underlying syndrome vs. isolated genetic hearing loss.

## Introduction

Every 2–3 children out of 1,000 in the United States are born with hearing loss (HL), making it the most common congenital sensory deficit in humans (1). Sensorineural hearing loss predominates congenital hearing loss, with the causes of HL broadly divided into genetic vs. non-genetic or acquired factors. Over the past 25 years the continual advancement of technology and accuracy of diagnostic testing has revealed genetic etiology for HL occurrences in prelingual children to be as high as 60% (2).

Despite this advancement in knowledge of genetic causes, there is still limited consensus on management of pediatric patients with hearing loss (3). The primary goals of management of pediatric patients with HL are timely and proper diagnosis and determining appropriate aural rehabilitation. The aim of achieving these goals is to optimize communication and language development in the child with hearing loss.

Genetic testing may provide insight into management of hearing loss itself or provide guidance of when to consider additional congenital anomalies in association with a genetic syndrome. For example, individuals with sensorineural hearing loss who have Usher syndrome are at risk for retinopathy and this should be followed closely. In addition, monogenic causes of hearing loss may present with a spectrum within a family. Therefore, diagnosis in a child with profound hearing loss may prompt testing in other family members who have progressive hearing loss or allow for treatment of other affected family members at a younger age.

In this article, we will review an approach to genetic testing for individuals with hearing loss and then discuss the evidence regarding management as a guide to optimizing future practice.

## Pediatric Hearing Loss Overview

The American Academy of Pediatrics' Joint Committee on Infant Hearing is a leader in endorsing early detection of and intervention for infants with hearing loss. They proposed the 1–3–6 guideline for screening and interventions for pediatric HL. This is designed to maximize the outcome for infants who are deaf or hard of hearing and recommends that all infants should be screened for hearing loss no later than 1 month of age. Infants that do not pass their screening are recommended to have a comprehensive audiological evaluation [auditory brainstem response (ABR) testing] no later than 3 months of age, and infants that do have confirmed hearing loss should receive appropriate intervention no later than 6 months. Intervention should be from health care and education professionals with expertise in hearing loss and deafness in infants and young children (4).

Infants admitted to the neonatal intensive care unit (NICU) require more in-depth screening based on days of admission. NICU infants admitted to the hospital for more than 5 days should have ABR included as part of their screening (4). Regardless of newborn hearing-screening outcome, all infants should continue to have ongoing monitoring for developmentally appropriate communication skills and auditory behaviors.

## Non-Genetic Etiology Work-Up

Clinicians should establish potentially reversible causes of hearing loss as soon as possible. Half of all the non-genetic causes of HL are attributed to infectious disease. TORCHES [toxoplasmosis, rubella, cytomegalovirus (CMV), herpes, and syphilis] infections are known risk factors for SNHL. Out of these infectious causes, congenital CMV is the most common cause of non-hereditary SNHL in childhood. Most estimates from studies conducted in Europe, the United States, and Japan show the prevalence of congenital CMV varies from 0.2 to 2.0%, but much higher in developing countries at 6–14% (5). CMV infection is congenital if diagnosed within 21 days postnatally. CMV infection in infants if acquired postnatally is not associated with SNHL (3). At our institution, we test for congenital CMV using saliva polymerase chain reaction (PCR) and if this is positive confirmation is with urine PCR. Treatment for congenital CMV in children with isolated SNHL is still not fully elucidated. Antiviral therapy, particularly with valganciclovir, is the most commonly used medication for treatment though the benefits in the literature are mixed (3). When a patient is diagnosed with congenital CMV with isolated SNHL, infectious disease consultation is recommended to review the risks and benefits of antiviral therapy and consider treatment.

Overall in the literature, use of CT in children with hearing loss ranges from 20 to 70% of the time (6). It is superior to MRI in delineating bony anatomy and, therefore, useful in understanding conductive or mixed hearing loss, and CT has also been shown to have a higher yield in identifying enlarged vestibular aqueducts and cochlear anomalies (7). Though it is less expensive and faster than MRI, it does expose children to ionizing radiation, so this risk should be considered when deciding on diagnostic imaging. MRI is preferred for evaluating the cochlear nerve, and the brain and nerve tumors (such as vestibular schwannoma). MRI frequently requires sedation or general anesthesia to perform and the risks associated with this also need to be considered when choosing an imaging modality. When it comes to surgical planning, particularly for possible cochlear implantation, CT or MRI can be used and usually up to the surgeon's discretion. The IPOG study group had 100% agree or partially agree that children who are cochlear implant candidates with profound hearing loss may benefit from CT or MRI to assess for cochlear dysplasia and cochlear nerve hypoplasia/aplasia (8).

If the pediatric HL is considered to be non-genetic and non-infectious in etiology, additional testing should be based on clinical exam findings, imaging, and medical and family history. A “shotgun” approach to obtain an etiologic diagnosis for pediatric HL has been shown to have very low diagnostic yield and to unnecessarily increase cost (9). However, certain investigations are value-additive.

## Genetic Work-Up

Any infant or child diagnosed with bilateral congenital HL without a known etiology (e.g., infectious) requires a genetics consultation. Comprehensive genetic testing has the highest diagnostic yield of any single test for bilateral SNHL and up to 60% of cases of congenital HL are due to a genetic etiology (8, 10). Originally the only available genetic testing for hearing loss was single-gene testing, most commonly for *GJB2* and/or *GJB6* genes (10). Comprehensive genetic testing (CGT), by using massively parallel sequencing or next generation sequencing (NGS), now improves the genetic diagnostic yield by multiple orders of magnitude over single-gene testing and is becoming the new standard of care due to decreased cost of sequencing and avoidance of multiple tests and appointments (10, 11). IPOG consensus recommendations also state this with 84% agreement or in partial agreement that in the setting of CPG, single-gene testing is of low diagnostic yield and should only be offered as part of an initial workup if a known family history exists. All surveyed agree or partially agree that single-gene testing should be considered if CGT is not available (8). It is important to note that a negative genetic test does not rule out a genetic etiology, as the gene of interest may not have been included due to lack of known role in HL to date or the sensitivity of the test may not have picked up the causative mutation (such as intronic mutations). Whole genome sequencing can be used as well to detect lesser known hearing loss mutations not covered in the NGS testing. Table 1 lists some of the more commonly available commercial NGS tests in the United States market, which all evaluate for syndromic and non-syndromic causes for congenital hearing loss.

**Table 1:** NGS tests in US.

	**Laboratory**	**Turn Around Time**	**# of Genes Analyzed**
OtoSeq	Cincinnati children's molecular genetics laboratory	8 weeks	23
Fulgent comprehensive hearing loss NGS panel	Fulgent genetics	3–5 weeks	179
OtoGenetics comprehensive deafness gene testing	OtoGenetics corporation	4 weeks	129
OtoSCOPE	Iowa molecular otolaryngology and renal research laboratories	6 weeks	152
Emory hearing loss expanded panel	Emory genetics laboratory	12 weeks	92
OtoGenome	Laboratory for molecular medicine, partners healthcare personalized medicine	8–12 weeks	110

Genetic etiologies for congenital HL can be divided into syndromic or non-syndromic SNHL (NSSNHL). Approximately 70% of cases of genetic-related hearing loss are NSSNHL, which are often contributed to a single gene mutation. The remaining 30% syndromic SNHL cases are often accompanied by other physical abnormalities or systemic clinical manifestations. Table 2 provides a list of the most common syndromic causes of hearing loss. The number of known NSSNHL related to disease-causing genetic variants continues to grow as the technology and capability of genetic testing has expanded. However, the number of known syndromic SNHL genetic causes has remained relatively stable over the past 10 years with ~500 known syndromes associated with HL (12, 13).

**Table 2:**
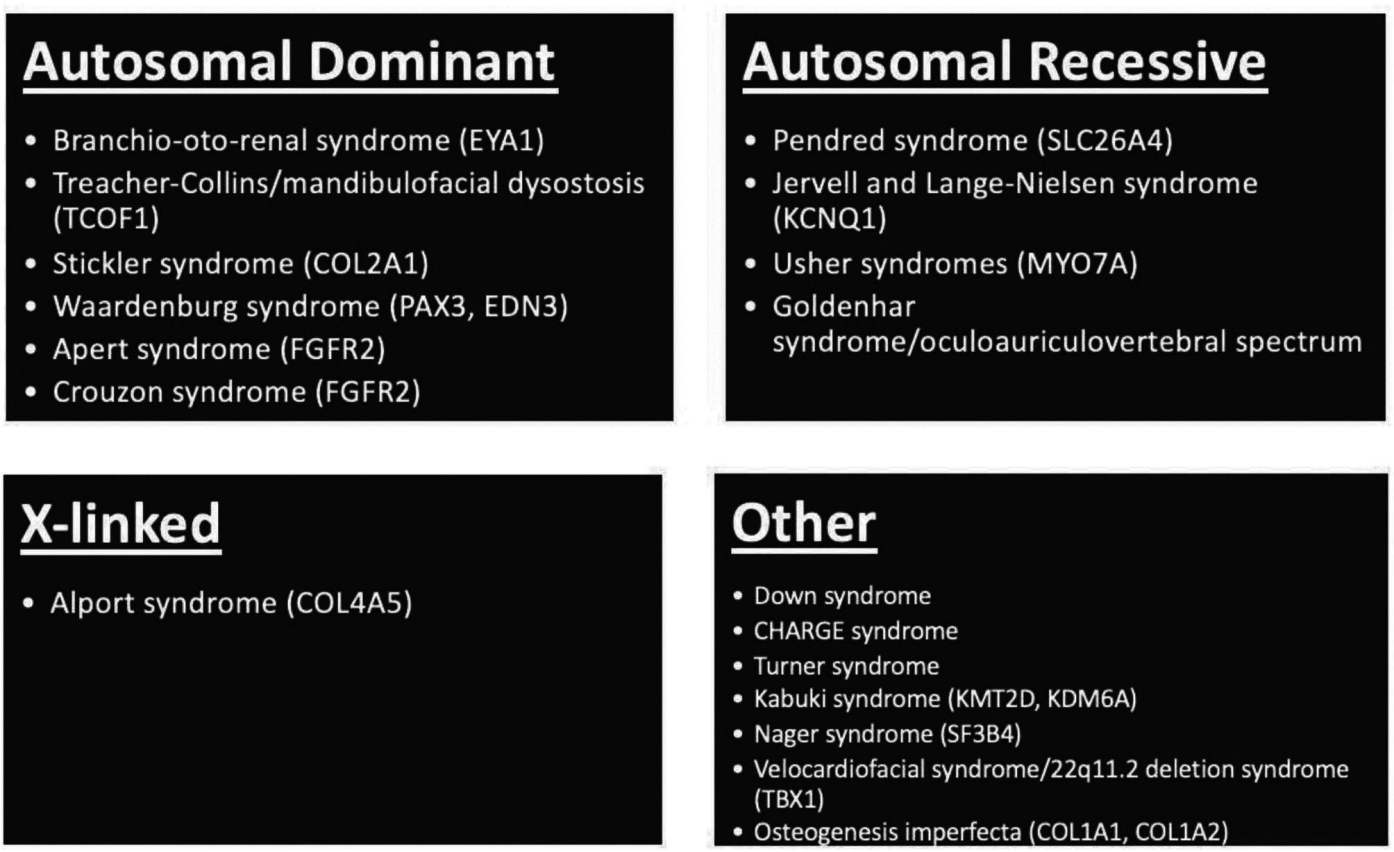
List of syndromic HL.

The Clinical Genome Resource (ClinGen), a National Institutes of Health (NIH)-funded initiative has built a central resource to provide a semiquantitative framework to assign clinical validity to the gene-disease relationships in the hundreds of genes reported in the literature associated with hearing loss. The gene-disease pairs are classified by either being definitive, strong, moderate, limited, disputed, or refuted. For the hearing loss associated genes ClinGen applied the clinical validity framework on 142 genes associated with non-syndromic and syndromic hearing loss that are included on panels from 17 diagnostic testing laboratories from around the world (14). The clinical validity classifications for these genes are publicly available at: https://search.clinicalgenome.org/kb/gene-validity.

Genetic diagnosis may help practitioners provide anticipatory guidance to families regarding other involved organ systems. This can provide significant insight into the patient's care. All children confirmed to have SNHL should have a vision screen either by their pediatrician, optometrist, or ophthalmologist as part of their work-up, given the 2–3-fold increased risk of ocular abnormalities in children with non-syndromic SNHL (8). A recent study demonstrated that the majority of ocular abnormalities in children with hearing loss can be identified with today's standard screening methods (15). If a child has profound bilateral SNHL and there is a family history of cardiac arrhythmia or sudden death of unknown etiology, then it is also recommended to obtain an electrocardiogram to rule out Jervell and Lange-Nielsen syndrome.

In the NSSNHL group, 75–80% are inherited in an autosomal recessive pattern, with 15–24% autosomal dominant, 1–2% X-linked, and <1% mitochondrial (2, 13, 16). Abnormalities of *GJB2* are the most common cause of HL in infants, and account for ~50% of the autosomal recessive HL patients. Since only half of the responsible mutations of *GJB2* are biallelic, there are a significant number with only one mutation therefore screening for only this gene is insufficient (2). *GJB2* is located on chromosome 13q and encodes for the protein connexin 26, while *GJB6* is also located on the same chromosome and lies adjacent to it at the DFNB1 locus. Connexin 30 which forms gap junctions with Connexin 26 is encoded by *GJB6*. Thus, NSSNHL loss can also occur with a mutation in *GJB2* on one allele coupled with a deletion in *GJB6*, and explains the HL in patients when *GJB2* is not inherited in a biallelic fashion (2). Over 110 disease-causing variants in *GJB2* and at least 2 deletions in *GJB6* have been reported (2, 17, 18). It should be noted that the most common *GJB2* disease-causing variants that are found in the Caucasian populations, do not play a large role in other races, particularly those of African descent (10, 19–21). The second most common genetic cause of bilateral SNHL is a mutation in the *STRC* gene on 15q15.3, which can be from partial or complete gene deletions, copy number variants (CNV), or point mutations of the gene (2, 22).

The diagnostic yield of genetic testing in children with unilateral SNHL ranges from 1 to 5% in the literature (23, 24). However, it can have huge implications on the patient's care including screening and should be considered early on in the work up. In particular, all individuals with bilateral congenital sensorineural hearing loss should be sent for genetics work up. In addition, a family history of hearing loss should prompt referral.

## Imaging In Hearing Loss

**Figure 1:**
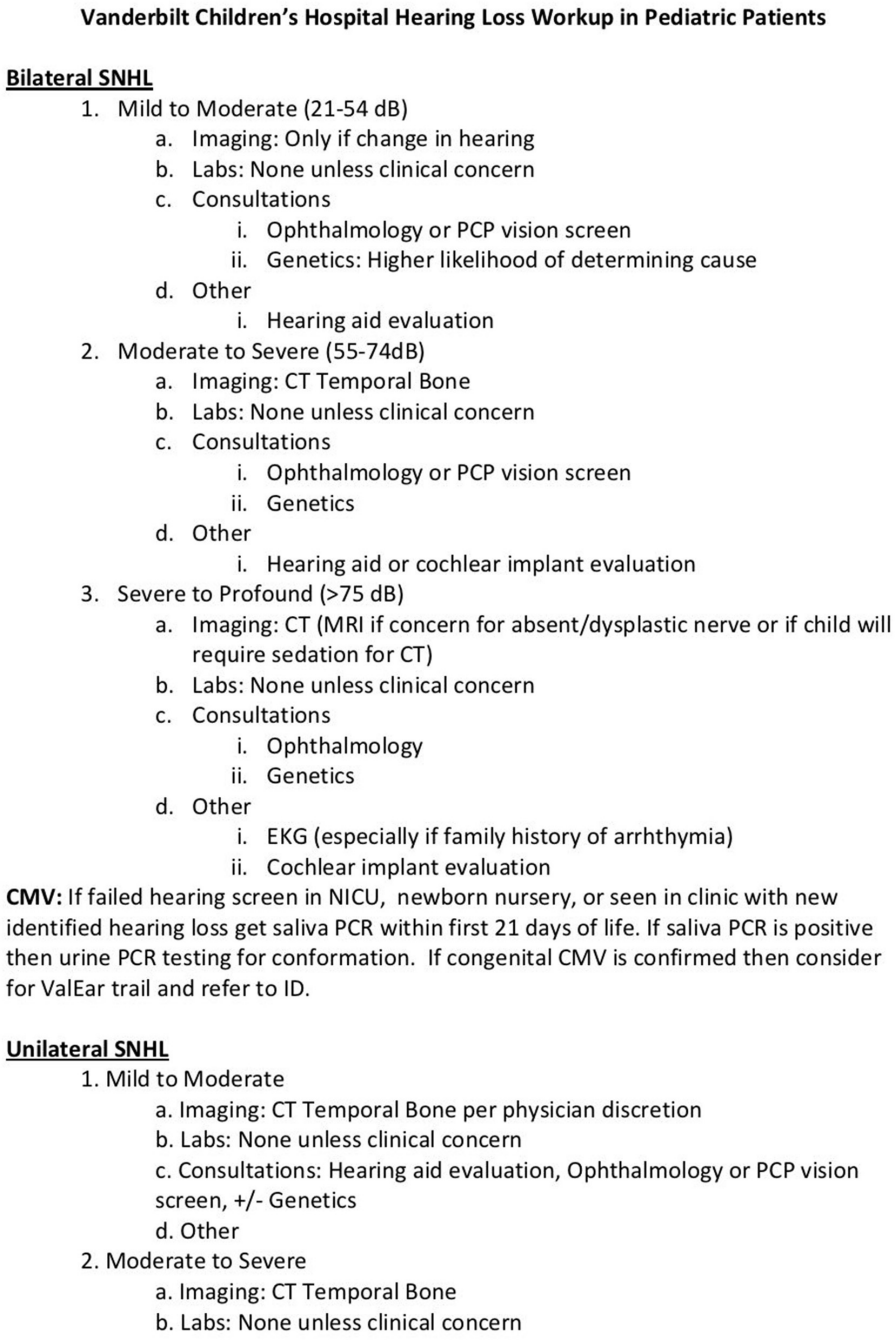
Vanderbilt Children's Hospital hearing loss protocol.

Radiographic imaging can play an important role in diagnosis in genetic or non-genetic etiologies for pediatric HL. See Figure 1 for our institution's protocol for imaging depending on the degree of hearing loss, unilateral vs. bilateral, or if post-meningitis. Whether a computed tomography (CT) of the temporal bones or magnetic resonance imaging (MRI) is ordered, children with more severe SNHL are more likely to have an abnormality on imaging (3). Currently, there is neither consensus on the best temporal bone imaging modality nor on the timing of obtaining a scan. However, an international working group of pediatric otolaryngologists—IPOG—expressed a majority opinion that temporal bone imaging should not be performed during the neonatal period unless indicated for other reasons (i.e., brain MRI) (8). The same IPOG publication sought to obtain consensus surrounding pediatric hearing loss. In a survey, 76% IPOG members agree or partially agree that temporal bone imaging is of low diagnostic yield when routinely employed in the setting of symmetric bilateral hearing loss. Note they did not detail this or break it down into severity of the hearing loss. All agree or partially agree that for unilateral, asymmetric or mixed loss, the diagnostic yield of imaging is higher and should be considered. All surveyed also agree or partially agree that children that are cochlear implant candidates with profound hearing loss may benefit from CT or MRI.

## Conclusion

Identifying a genetic etiology has been shown to have many clinical benefits as well as psychosocial and emotional benefits for patient families. Obtaining a genetic diagnosis has been shown to improve parental psychological well-being, mostly by alleviating guilt and accelerating involvement in rehabilitation (10, 25–27). It also allows for parental education of future medical or educational needs and potential risk of hearing loss in future offspring (10). If definitive results are found on genetic testing, this may also preclude the need for imaging and further costs (24). Finally, for children requiring rehabilitation through cochlear implantation, genetic testing may help benchmark expected outcomes against specific lesions that have been characterized by busy sequencing centers that aggregate both genotypic and phenotypic (audiometric and speech-related) information.

## Author Contributions

RB responsible for writing manuscript draft, editing, and figure/table creation. JD, CW, and FV responsible for writing and editing manuscript and creation of figure protocol. All authors contributed to the article and approved the submitted version.

## Conflict of Interest

The authors declare that the research was conducted in the absence of any commercial or financial relationships that could be construed as a potential conflict of interest.
