# Supplementary material for: Genetic and Non-genetic Workup for Pediatric Congenital Hearing Loss
Source: Front Pediatr. 2021 Mar 22;9:536730. doi: 10.3389/fped.2021.536730 (PMC8020033; doi:10.3389/fped.2021.536730)
Supplement: Supplementary file 1 [file Data_Sheet_1.PDF]

**Vanderbilt Children's Hospital Hearing Loss Workup in Pediatric Patients**

**Bilateral SNHL**

1. Mild to Moderate (21-54 dB)
    a. Imaging: Only if change in hearing
    b. Labs: None unless clinical concern
    c. Consultations
        i. Ophthalmology or PCP vision screen
        ii. Genetics: Higher likelihood of determining cause
    d. Other
        i. Hearing aid evaluation
2. Moderate to Severe (55-74dB)
    a. Imaging: CT Temporal Bone
    b. Labs: None unless clinical concern
    c. Consultations
        i. Ophthalmology or PCP vision screen
        ii. Genetics
    d. Other
        i. Hearing aid or cochlear implant evaluation
3. Severe to Profound (>75 dB)
    a. Imaging: CT (MRI if concern for absent/dysplastic nerve or if child will require sedation for CT)
    b. Labs: None unless clinical concern
    c. Consultations
        i. Ophthalmology
        ii. Genetics
    d. Other
        i. EKG (especially if family history of arrhthymia)
        ii. Cochlear implant evaluation

**CMV:** If failed hearing screen in NICU, newborn nursery, or seen in clinic with new identified hearing loss get saliva PCR within first 21 days of life. If saliva PCR is positive then urine PCR testing for conformation. If congenital CMV is confirmed then consider for ValEar trail and refer to ID.

**Unilateral SNHL**

1. Mild to Moderate
    a. Imaging: CT Temporal Bone per physician discretion
    b. Labs: None unless clinical concern
    c. Consultations: Hearing aid evaluation, Ophthalmology or PCP vision screen, +/- Genetics
    d. Other
2. Moderate to Severe
    a. Imaging: CT Temporal Bone
    b. Labs: None unless clinical concern

c. Consultations: Hearing aid evaluation, Ophthalmology or PCP vision screen, +/- Genetics
d. Other

3. Severe to profound
    a. Imaging: CT Temporal Bone
    b. Labs: None unless clinical concern
    c. Consultations: Hearing aid evaluation, Ophthalmology or PCP vision screen, +/- Genetics
    d. Other

**<u>Bilateral Conductive without evidence of middle ear disease</u>**

1. Imaging: CT
2. Bone Anchored Implant Evaluation
3. Microtia/Atresia Clinic if appropriate

**<u>Unilateral Conductive without evidence of middle ear disease</u>**

1. Imaging: CT
2. Bone Anchored Implant Evaluation
3. Microtia/Atresia Clinic if appropriate

**<u>Post Meningitis:</u>**

1. Hearing screen/evaluation while in the hospital
2. If patient PASSES initial screen, return for audiogram in 1 month.
    a. Bacterial - If hearing is WNL at 1 month visit, recommend follow-up at 3 mo. Will recommend follow-up at 3 month intervals until 1 year from illness.
    b. Viral – If hearing is WNL at 1 month visit, recommend follow-up at 6 months and 12 months from illness. Consider yearly screening to monitor.
3. If patient FAILS initial screen, formal audiogram vs. ABR asap.
    a. If hearing WNL, recommend follow-up as explained above.
    b. If hearing loss:
        1. ENT evaluation ASAP
        2. Consider Imaging MRI and CT (MRI is more sensitive for early Labryinthitis)
        3. Hearing aid or CI evaluation

**Speech/Language Eval**:

Initiate referral for speech/ language evaluation at initial consultation for hearing loss. Evaluation is recommended for all children with bilateral hearing loss of any degree. Referrals for children with unilateral hearing loss should be made on a case-by-case basis informed partly by family motivation, speech concerns, etc.
